# Composite indices of insulin resistance and sensitivity versus matched component-variable models for incident diabetes prediction

**DOI:** 10.3389/fendo.2026.1957473

**Published:** 2026-10-08

**Authors:** Qiujun Liu, Canyu Liu, Yuanhao Lv, Yanxuan Liu

**Affiliations:** 1 Department of Endocrinology, the First Affiliated Hospital of Henan Medical University, Xinxiang, China; 2 Department of Digestive Endoscopy Center, the First Affiliated Hospital of Henan Medical University, Xinxiang, China; 3 Department of Pathology, Henan Medical University, Xinxiang, China; 4 Department of Medical Genetics, the First Affiliated Hospital of Henan Medical University, Xinxiang, China

**Keywords:** diabetes, insulin resistance, METS-IR, risk prediction, SPISE, TyG

## Abstract

**Background:**

The triglyceride–glucose (TyG) index, single-point insulin sensitivity estimator (SPISE), and metabolic score for insulin resistance (METS-IR) are widely used non-insulin-based surrogate indices. Whether their fixed formulas predict diabetes better than models retaining the same component measurements remains uncertain.

**Methods:**

We analyzed 112,070 participants from the RICH cohort and externally validated frozen RICH models in 15,453 participants from the NAGALA cohort. Cox models compared each index with a matched model containing the same component variables separately; all models included age, sex, systolic blood pressure, and alanine aminotransferase. Internal validation used 1,000 bootstrap resamples and leave-one-center-out validation. External paired AUC differences were estimated using 1,000 participant-level bootstrap resamples. Additional analyses evaluated the contribution of FPG and repeated the index–component comparisons with FPG retained separately in both models. Likelihood-ratio tests evaluated formula constraints.

**Results:**

Higher TyG and METS-IR and lower SPISE were nonlinearly associated with incident diabetes. In RICH, component-variable models had higher optimism-corrected 3-year AUCs than the TyG, SPISE, and METS-IR models by 0.051 (95% CI, 0.037–0.065), 0.009 (0.004–0.016), and 0.059 (0.047–0.073). In NAGALA, these differences were 0.072 (0.040–0.106), −0.006 (−0.021 to 0.010), and 0.046 (0.014–0.079), respectively. These models underestimated 3-year diabetes risk (observed-to-expected ratios, 1.25–2.52). Entering FPG separately in both models narrowed differences. In NAGALA, no clear difference was observed for TyG or SPISE, whereas the METS-IR model had a higher AUC than its matched component-variable model by 0.013 (0.004–0.022). Conventional likelihood-ratio tests favored freely estimated formula-scale coefficients for all three indices (all Holm-adjusted *P* < 0.001), although SPISE inference was sensitive to center clustering. Freely estimating these coefficients improved AUC in both cohorts only for TyG. The comparison of TyG with FPG plus triglycerides showed consistent results in the expanded sample. Component-variable advantages for TyG and METS-IR persisted with splines and at 2.5 years.

**Conclusion:**

Component-variable models showed higher discrimination than the matched TyG and METS-IR models, whereas the small difference for SPISE was not reproduced externally. These differences were reduced when FPG was retained separately. Freely modeling log-transformed FPG and triglycerides improved discrimination over TyG in both cohorts.

## 1 Introduction

Diabetes mellitus has become one of the most pressing global health challenges. The International Diabetes Federation estimated that 589 million adults aged 20–79 years were living with diabetes worldwide in 2024, with this number projected to reach 853 million by 2050 (1). Diabetes develops gradually through a prolonged phase of metabolic dysfunction. Identifying high-risk individuals before disease onset is therefore essential for diabetes prevention.

Insulin resistance is central to the pathogenesis of type 2 diabetes. The hyperinsulinemic–euglycemic clamp is the reference standard for measuring insulin resistance (2), but its complexity, cost and time requirements make it unsuitable for population-based screening (3). These limitations have prompted the development of non-insulin-based surrogate indices, including The triglyceride-glucose index (TyG), single point insulin sensitivity estimator (SPISE), and metabolic score for insulin resistance (METS-IR), which are calculated from routinely available measurements such as fasting plasma glucose (FPG), triglycerides (TG), high-density lipoprotein cholesterol (HDL-C) and body mass index (BMI) (4–6). Previous cohort studies have linked higher TyG and METS-IR or lower SPISE to an increased risk of incident diabetes and have reported moderate discriminatory performance for these indices (7–9).

Previous studies have primarily compared these surrogate indices using measures of association or discrimination, thereby providing valuable evidence on their relative performance. A complementary question is whether the prespecified index formulas retain the predictive information contained in their constituent measurements as effectively as models that include those measurements separately. Composite indices provide a concise summary of metabolic status, whereas component-variable models allow the contribution of each measurement to be estimated specifically for the outcome and population under study. In a previous study, FPG alone achieved a higher AUC for incident diabetes than either TyG or TyG-BMI (10), suggesting that the predictive performance of composite indices relative to their underlying measurements warrants further evaluation.

Whether these indices provide predictive advantage over models retaining the same component variables remains unclear. Therefore, we used two independent East Asian health-screening cohorts to compare the 3-year predictive performance of TyG, SPISE and METS-IR with that of matched models containing their corresponding component variables. Additional analyses examined whether the indices added predictive information beyond FPG and whether freely estimating formula-scale component weights improved prediction.

## 2 Materials and methods

### 2.1 Datasets

This retrospective cohort study was based on two publicly available health examination datasets from East Asia. Data from the Chinese Rich Healthcare Group (RICH) cohort were used as the primary analysis and model-development cohort. The cohort was established using records from adults attending 32 health examination centers in 11 Chinese cities between 2010 and 2016. Chen et al. described the original cohort and deposited the de-identified analytic dataset in the Dryad Digital Repository (doi: 10.5061/dryad.ft8750v) (11). External validation was performed using data from the NAfld in the Gifu Area, Longitudinal Analysis (NAGALA) cohort. This cohort was established among adults undergoing repeated health examinations at Murakami Memorial Hospital in Gifu, Japan. The study design was originally reported by Okamura et al., who subsequently made the de-identified analytic dataset available through Dryad (doi: 10.5061/dryad.8q0p192) (12). The original RICH study was approved by the Rich Healthcare Group Review Board, whereas the NAGALA study was approved by the Ethics Committee of Murakami Memorial Hospital. The present study involved secondary analysis of de-identified data and included no additional participant contact or intervention. Reporting followed the STROBE and TRIPOD+AI statements (13, 14).

### 2.2 Study population

The publicly available RICH and NAGALA datasets included 211,833 and 15,464 participants, respectively. In this study, we selected participants with a baseline fasting plasma glucose (FPG) concentration >0 and<6.1 mmol/L, and excluded those with missing data on age, sex, follow-up time, or diabetes outcome, as well as those with missing or non-positive values for systolic blood pressure (SBP), alanine aminotransferase (ALT), triglycerides (TG), high-density lipoprotein cholesterol (HDL-C), or body mass index (BMI). The final analytical cohorts comprised 112,070 participants from RICH and 15,453 from NAGALA (**Figure 1**).

**Figure 1:**
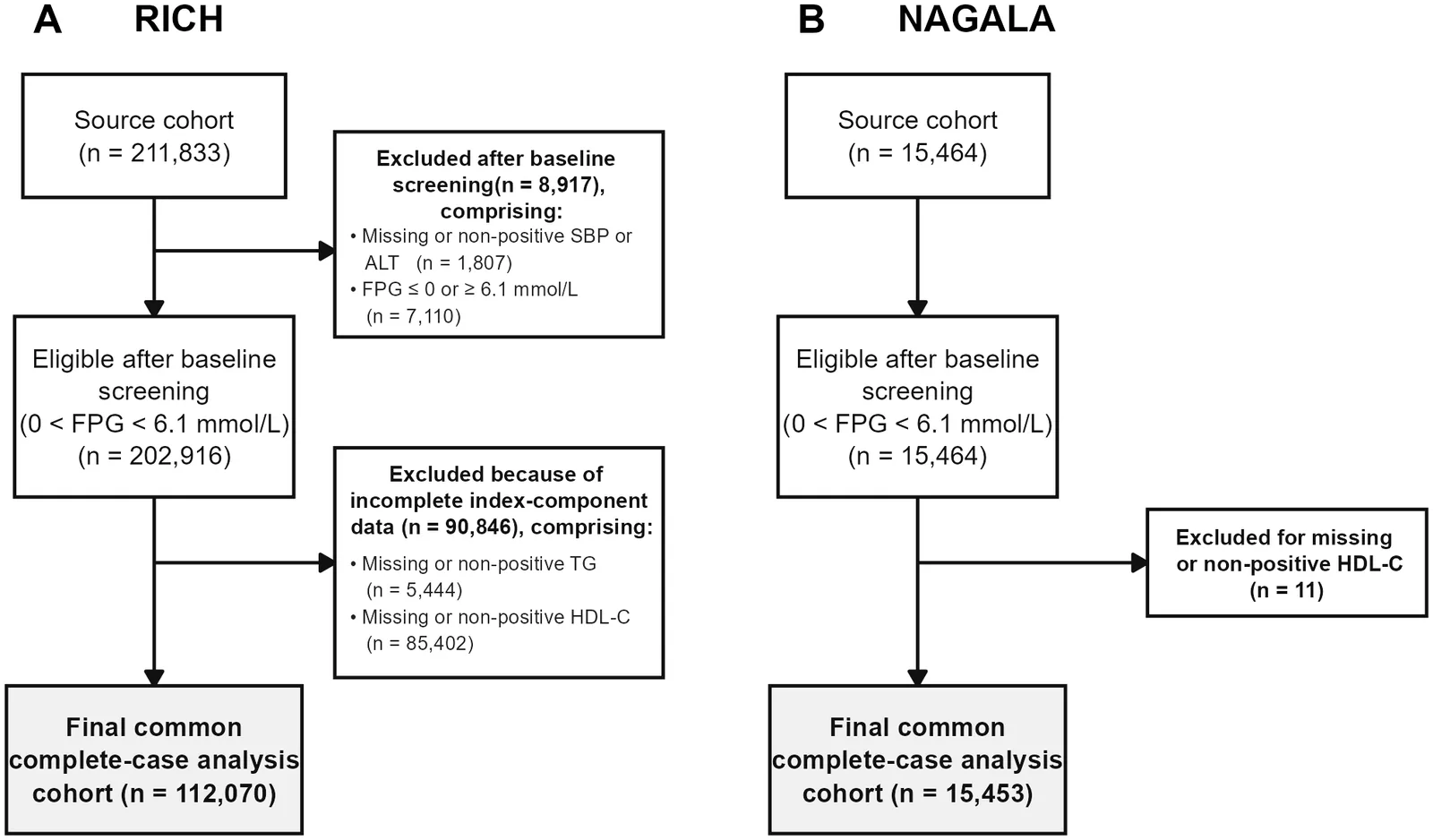
Flowchart of participant selection. **(A)** Selection of participants from the RICH cohort. **(B)** Selection of participants from the NAGALA cohort.

### 2.3 Data collection and definitions

Baseline age, sex, SBP, ALT, FPG, TG, HDL-C, and BMI were obtained from the health examination records. BMI was calculated as body weight in kilograms divided by height in meters squared. Blood samples were collected after an overnight fast and analyzed according to the protocols of the original studies (11, 12). FPG, TG, and HDL-C were expressed in mmol/L in both cohorts. Follow-up time was recorded in years in RICH and converted from days to years in NAGALA using 365.25 days per year.

### 2.4 Calculation of insulin resistance and sensitivity indices

Before calculation, FPG was converted from mmol/L to mg/dL by multiplying by 18.0182; TG and HDL-C were converted by multiplying by 88.57 and 38.67, respectively. TyG, SPISE, METS-IR were calculated using the following published formulas (4–6):


$$TyG=ln\left[\frac{TG\left(mg/dL\right)\times FPG\left(mg/dL\right)}{2}\right]$$

$$SPISE=\frac{600\times HDL-C{\left(mg/dL\right)}^{0.185}}{TG{\left(mg/dL\right)}^{0.2}\times BMI{\left(kg/m^{2}\right)}^{1.338}}$$

$$METS-IR=\frac{\ln\left[2\times FPG\left(mg/dL\right)+TG\left(mg/dL\right)\right]\times BMI\left(kg/m^{2}\right)}{\ln\left[HDL-C\left(mg/dL\right)\right]}$$

Higher TyG and METS-IR values indicate greater insulin resistance, whereas lower SPISE values indicate greater insulin resistance. For consistent interpretation, SPISE was reverse-coded before standardization so that higher standardized values represented greater insulin resistance for all three indices. Means and standard deviations estimated in the RICH cohort were used for standardization and were applied unchanged to the NAGALA cohort.

### 2.5 Outcome definition

The outcome was incident diabetes, as defined in the original studies. In the RICH cohort, diabetes was identified by a fasting plasma glucose concentration of ≥7.0 mmol/L or self-reported diabetes. Participants were followed until the date of diabetes diagnosis or their last recorded health examination, whichever occurred first. In the NAGALA cohort, incident diabetes was identified by a fasting plasma glucose concentration of ≥7.0 mmol/L, an HbA1c level of ≥6.5%, or self-reported diabetes during follow-up. Event status and follow-up time were obtained directly from the publicly released datasets (11, 12).

### 2.6 Statistical analysis

Continuous variables with a normal or approximately normal distribution are presented as mean ± standard deviation, skewed continuous variables as median (Q1, Q3), and categorical variables as number and percentage. Between-cohort comparisons used Welch’s *t*-test, the Wilcoxon rank-sum test or Pearson’s chi-square test, as appropriate, and standardized mean differences were calculated. Quartiles of TyG, SPISE and METS-IR were defined in RICH. Cumulative diabetes incidence across quartiles was estimated as one minus the Kaplan–Meier survival function and compared using log-rank tests.

Associations with incident diabetes were evaluated in RICH using separate Cox models for each standardized index, adjusted for age, sex, systolic blood pressure and ALT. Models used all available follow-up, Efron handling of ties and center-clustered robust standard errors; proportional hazards were assessed using Schoenfeld residuals. Nonlinear associations were examined using restricted cubic splines with knots at the 5th, 35th, 65th and 95th percentiles (15). Stand-alone discrimination of each index was evaluated using the 3-year cumulative/dynamic AUC (16). The base model included age, sex, systolic blood pressure and ALT; each index model was compared with a model containing the same base covariates, with components entered linearly on their original scales.

Three-year AUC and Brier score were estimated using inverse probability of censoring weights. Calibration was assessed using the O/E ratio, calibration intercept and slope, and flexible calibration curves. Unless otherwise specified, RICH performance and paired model differences were optimism-corrected using 1,000 bootstrap resamples. Leave-one-center-out validation was also performed. For external validation, all RICH coefficients, standardization parameters and baseline risk were applied unchanged to NAGALA without refitting or recalibration. External paired differences and confidence intervals were estimated using 1,000 participant-level bootstrap resamples.

The contribution of FPG to the base model, the incremental value of each index beyond FPG, and index–component comparisons with FPG retained separately in both models were evaluated. Formula-constrained and nested freely weighted models on the TyG, −ln(SPISE) and ln(METS-IR) scales were compared using conventional likelihood-ratio tests based on the Cox partial likelihood (17). For METS-IR, only the outer weights were freed. *Post hoc* sensitivity checks used ordinary and center-clustered Wald tests of the same constraints. Center-level influence was assessed by omitting each center in turn and refitting both models. The three external matched index–component comparisons formed a prespecified Holm family for each performance metric. The FPG-conditioned, spline-based and earlier-horizon comparison sets were adjusted as separate Holm families, as were the index increments beyond FPG and the free-versus-constrained AUC comparisons. For each constraint-test method, *P* values were Holm-adjusted across the three indices. Baseline characteristics were compared between included and excluded RICH participants who were otherwise eligible for the TyG analysis to assess selection related to HDL-C availability. The TyG comparison was repeated in expanded samples requiring only variables needed for these models. The matched spline analysis was prespecified to be performed if nonlinearity was observed and used the four-knot specification above, with RICH-derived knots and model parameters applied unchanged to NAGALA. Because the source RICH cohort excluded participants with visit intervals shorter than 2 years, the 2-year prediction horizon could not be reliably evaluated; the earlier-horizon analysis was therefore performed at 2.5 years. For 3-year prediction, time-varying coefficients allowed ALT and, where separately included, HDL-C effects to differ before and after 2.5 years. RICH results for the matched-spline and time-varying-coefficient analysis were reported as apparent performance estimates. Decision-curve analysis assessed net benefit at 3-year risk thresholds of 0.5%–5.0% and was considered exploratory. All tests were two-sided, and *P<* 0.05 was considered statistically significant. Analyses were performed using R version 4.6.1.

## 3 Results

### 3.1 Baseline characteristics

The RICH development cohort included 112,070 participants from 15 examination centers, of whom 1,425 developed diabetes during follow-up, including 478 cases within 3 years. The NAGALA external validation cohort included 15,453 participants, among whom 373 developed diabetes, including 101 cases within 3 years. Median follow-up was 2.99 years (IQR, 2.13–3.94) in RICH and 5.39 years (IQR, 2.70–9.38) in NAGALA. Age and sex distributions were similar between cohorts (SMDs: 0.004 and 0.025, respectively).

Compared with RICH, participants in NAGALA had lower BMI, systolic blood pressure and triglyceride concentrations, but higher FPG and HDL-C concentrations. Correspondingly, NAGALA participants had lower TyG and METS-IR values and higher SPISE values. The largest between-cohort differences were observed for FPG, triglycerides, TyG and SPISE (SMD range, 0.521–0.579; **Table 1**).

**Table 1:** Baseline characteristics of the RICH and NAGALA cohorts.

Characteristic	RICH (n = 112,070)	NAGALA (n = 15,453)	*P* value	SMD
Age, years	43.67 ± 12.76	43.71 ± 8.90	0.613	0.004
Sex, n (%)			0.004	0.025
Female	52,392 (46.7%)	7,034 (45.5%)		
Male	59,678 (53.3%)	8,419 (54.5%)		
BMI, kg/m²	23.27 ± 3.27	22.12 ± 3.13	<0.001	0.359
SBP, mmHg	118.97 ± 16.44	114.49 ± 14.97	<0.001	0.285
ALT, U/L	18.00 (13.00, 27.00)	17.00 (13.00, 23.00)	<0.001	0.194
FPG, mmol/L	4.89 ± 0.54	5.16 ± 0.41	<0.001	0.561
TG, mmol/L	1.10 (0.75, 1.63)	0.73 (0.50, 1.12)	<0.001	0.521
HDL-C, mmol/L	1.38 ± 0.30	1.46 ± 0.40	<0.001	0.242
TyG	8.39 ± 0.60	8.03 ± 0.65	<0.001	0.567
SPISE	7.73 ± 2.10	9.11 ± 2.61	<0.001	0.579
METS-IR	33.61 ± 6.51	31.17 ± 6.50	<0.001	0.376

### 3.2 Risk stratification and dose–response associations in RICH

In the RICH cohort, cumulative diabetes incidence increased progressively across quartiles of TyG, SPISE and METS-IR (all log-rank *P* < 0.001; **Figure 2**). From the lowest-risk to highest-risk quartile, 3-year cumulative incidence increased from 0.17% to 1.49% for TyG, from 0.17% to 1.46% for SPISE, and from 0.17% to 1.46% for METS-IR.

**Figure 2:**
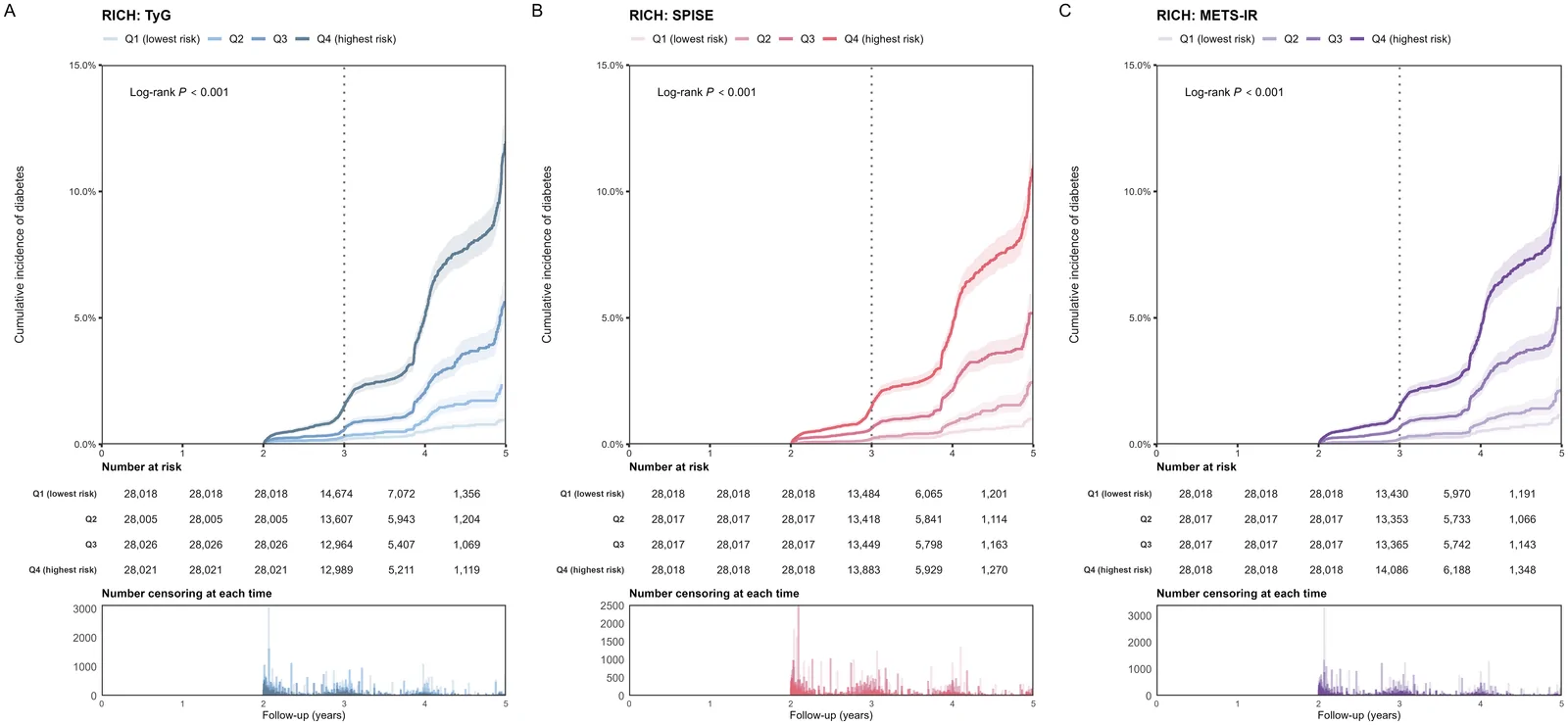
Cumulative incidence of diabetes by quartiles of TyG, SPISE and METS-IR in RICH. **(A)** TyG. **(B)** Reverse-coded SPISE. **(C)** METS-IR. Quartiles ranged from Q1 (lowest insulin resistance) to Q4 (highest insulin resistance). Curves show cumulative diabetes incidence estimated as one minus the Kaplan–Meier survival function; shaded areas indicate 95% confidence intervals. Numbers at risk and the numbers censored at each time are shown below each panel. The vertical dotted line indicates the prespecified 3-year prediction horizon. Differences across quartiles were assessed using log-rank tests.

After adjustment for age, sex, systolic blood pressure and ALT, all three indices were associated with incident diabetes. The HRs per 1-s.d. higher TyG and METS-IR were 1.76 (95% CI, 1.64–1.89) and 1.76 (1.64–1.88), respectively. For SPISE, each 1-s.d. decrease was associated with an HR of 2.27 (2.13–2.41; all *P<* 0.001; **Table 2**). Schoenfeld residual tests provided no evidence of non-proportionality for the TyG, reverse-coded SPISE or METS-IR terms (*P* = 0.977, 0.802 and 0.925, respectively). However, the global tests were significant (all *P<* 0.006), driven primarily by ALT; HDL-C also showed evidence of non-proportionality in the relevant component-variable models. Allowing time-varying coefficients for ALT and, where separately included, HDL-C produced little change in the 3-year index–component AUC differences.

**Table 2:** Adjusted associations of insulin resistance indices with incident diabetes in the RICH cohort.

Index	Contrast	Adjusted HR (95% CI)	*P* value
TyG	Per 1-SD higher TyG	1.76 (1.64, 1.89)	<0.001
SPISE	Per 1-SD lower SPISE	2.27 (2.13, 2.41)	<0.001
METS-IR	Per 1-SD higher METS-IR	1.76 (1.64, 1.88)	<0.001

Restricted cubic-spline analysis showed overall associations for all three indices (all *P* < 0.001). Nonlinearity was evident for TyG, SPISE and METS-IR (all *P* < 0.001). Risk increased more steeply at higher TyG and METS-IR values and at lower SPISE values (**Figure 3**). As standalone predictors in RICH, the three indices showed 3-year AUCs of 0.737 (TyG), 0.741 (SPISE) and 0.739 (METS-IR) (**Figure 4**).

**Figure 3:**
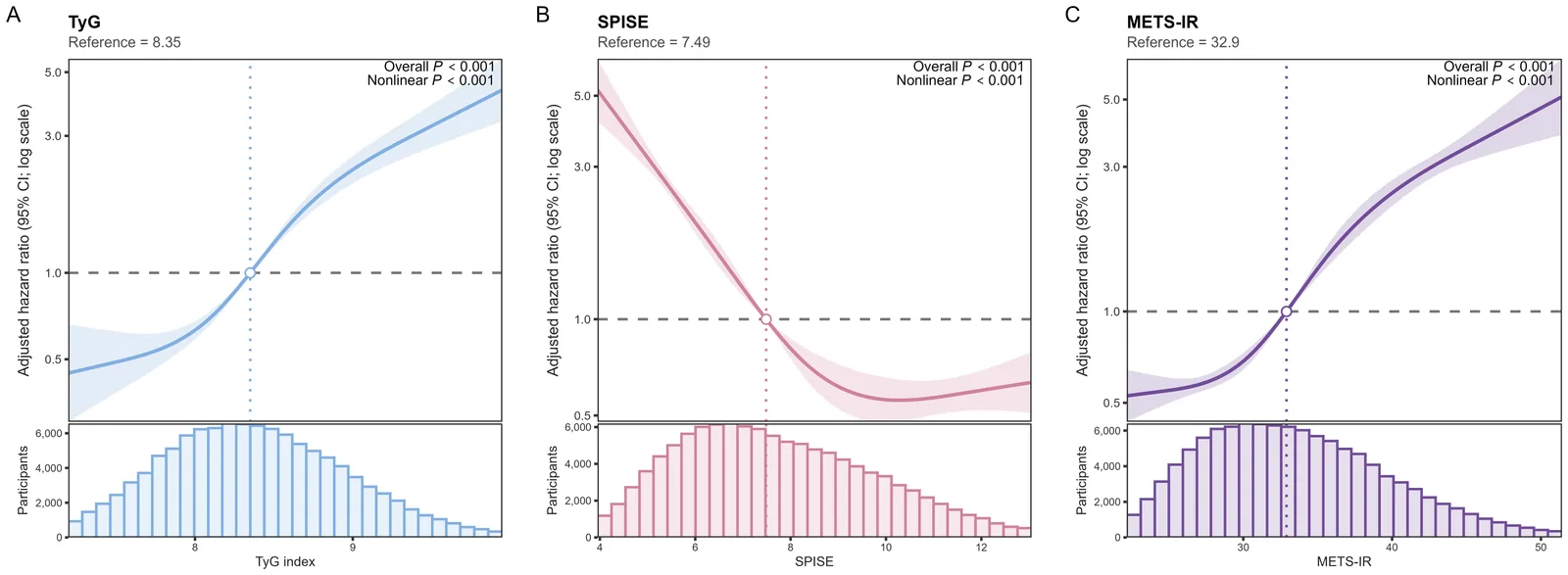
Nonlinear associations of TyG, SPISE and METS-IR with incident diabetes in RICH. **(A)** TyG. **(B)** SPISE. **(C)** METS-IR. Solid lines show adjusted hazard ratios and shaded areas show 95% confidence intervals estimated using restricted cubic splines. Models were adjusted for age, sex, systolic blood pressure and alanine aminotransferase. Horizontal dashed lines indicate a hazard ratio of 1, and vertical dotted lines indicate the reference values. Histograms show the distributions of the respective indices. SPISE was retained on its original scale to facilitate clinical interpretation.

**Figure 4:**
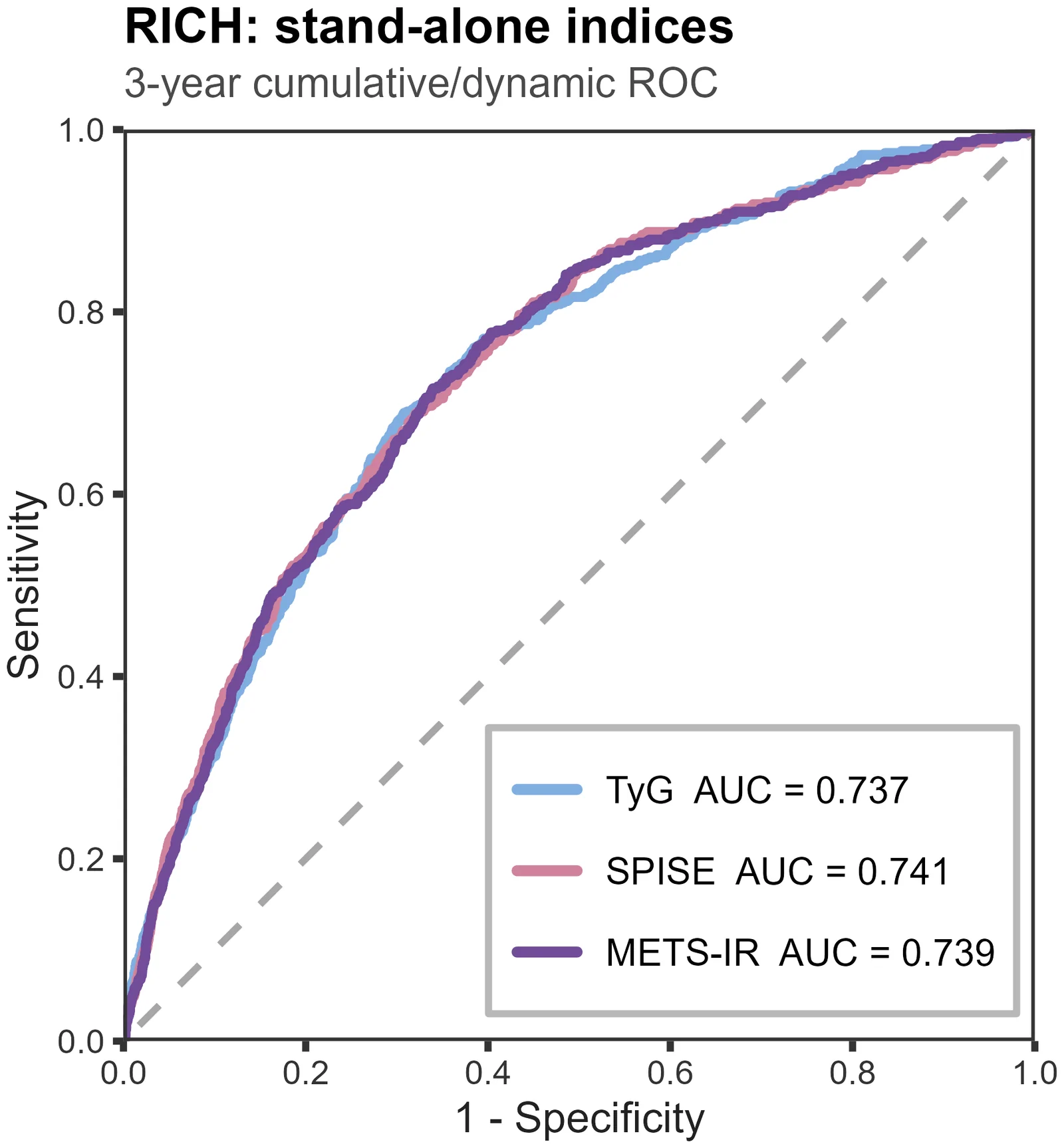
Stand-alone discrimination of TyG, reverse-coded SPISE and METS-IR for 3-year incident diabetes in RICH.

### 3.3 Comparative predictive performance and internal validation in RICH

After 1,000 bootstrap resamples, the optimism-corrected 3-year AUC was 0.769 (95% CI, 0.748–0.790) for the base model. The corresponding AUCs for the TyG, SPISE and METS-IR models were 0.802, 0.804 and 0.810, respectively, versus 0.854, 0.814 and 0.869 for their matched component-variable models. Relative to the corresponding index models, the matched component-variable models increased the optimism-corrected 3-year AUC by 0.051 (95% CI, 0.037–0.065) for TyG, 0.009 (0.004–0.016) for SPISE, and 0.059 (0.047–0.073) for METS-IR (**Table 3**). For TyG and METS-IR, the Brier scores were lower for the component-variable models; for SPISE, the difference was minimal. In pooled leave-one-center-out validation, the 3-year cumulative/dynamic AUC was 0.766 for the base model and increased to 0.801, 0.802 and 0.809 after the addition of TyG, SPISE and METS-IR, respectively (**Figure 5**).

**Table 3:** Model performance after 1,000 bootstrap resamples with optimism correction in the RICH cohort.

Model	3-year AUC (95% CI)	ΔAUC (95% CI)	3-year Brier	O/E	Calibration slope
Base	0.769 (0.748, 0.790)	−	0.0062	1.04	0.87
Base + TyG	0.802 (0.784, 0.822)	−	0.0062	1.03	0.91
Base + FPG + TG	0.854 (0.836, 0.870)	0.051 (0.037, 0.065)	0.0061	0.98	0.98
Base + SPISE	0.804 (0.785, 0.823)	−	0.0062	1.04	0.91
Base + BMI + TG + HDL-C	0.814 (0.795, 0.831)	0.009 (0.004, 0.016)	0.0062	1.04	0.93
Base + METS-IR	0.810 (0.791, 0.828)	−	0.0062	1.05	0.93
Base + FPG + TG + HDL-C + BMI	0.869 (0.853, 0.885)	0.059 (0.047, 0.073)	0.0061	0.98	0.99

ΔAUC was calculated as the AUC of the component-variable model minus that of the matched index model; positive values favor the component-variable model. Differences were calculated from unrounded estimates.

**Figure 5:**
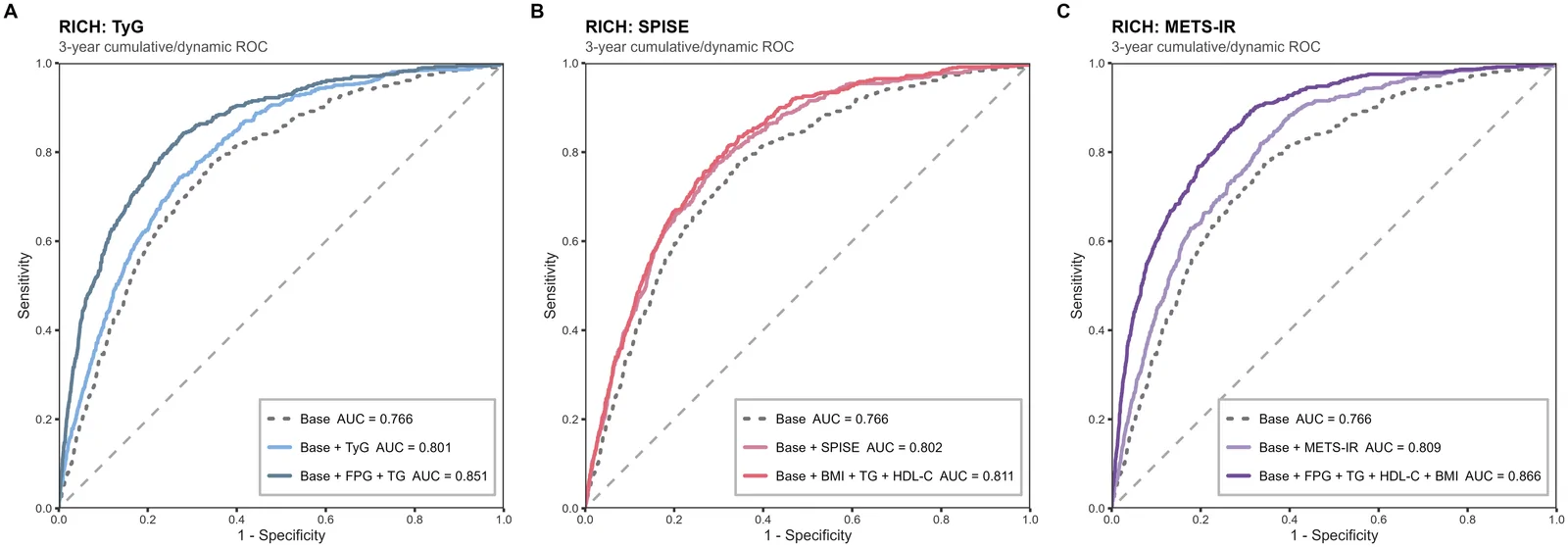
Time-dependent ROC curves at 3 years from leave-one-center-out validation in RICH. **(A)** Base, Base + TyG, and Base + FPG + TG. **(B)** Base, Base + reverse-coded SPISE, and Base + BMI + TG + HDL-C. **(C)** Base, Base + METS-IR, and Base + FPG + TG + HDL-C + BMI. The base model included age, sex, SBP and ALT. The diagonal dashed line represents discrimination expected by chance.

### 3.4 External validation in NAGALA

When applied unchanged to NAGALA, the component-variable models had higher 3-year AUCs than the corresponding TyG and METS-IR models, with paired differences of 0.072 (95% CI, 0.040–0.106) and 0.046 (0.014–0.079), respectively. The difference between SPISE and its component-variable model was small (component-variable model minus SPISE: ΔAUC, −0.006; 95% CI, −0.021 to 0.010; **Table 4**). Smooth calibration curves were predominantly above the identity line across the range containing most predictions, indicating systematic underprediction of 3-year diabetes risk in NAGALA (**Figure 6**). This was consistent with O/E ratios of 1.25–2.52 and positive calibration intercepts of 0.26–0.93. Calibration slopes ranged from 1.02 to 1.37, suggesting that the transported predictions were generally insufficiently dispersed. Recalibration would therefore be required before the RICH-derived probabilities could be used for absolute risk estimation in NAGALA. Exploratory decision-curve analysis of the unrecalibrated models are presented in **Supplementary Figure S1**.

**Table 4:** External validation in NAGALA without refitting.

Model	3-year AUC (95% CI)	ΔAUC (95% CI)	3-year Brier	O/E	Calibration slope
Base	0.731 (0.681, 0.777)	−	0.0078	1.78	1.20
Base + TyG	0.783 (0.736, 0.822)	−	0.0078	2.33	1.08
Base + FPG + TG	0.855 (0.817, 0.885)	0.072 (0.040, 0.106)	0.0077	1.25	1.37
Base + SPISE	0.790 (0.748, 0.830)	−	0.0077	2.52	1.02
Base + BMI + TG + HDL-C	0.784 (0.740, 0.825)	−0.006 (−0.021, 0.010)	0.0078	2.32	1.15
Base + METS-IR	0.807 (0.767, 0.845)	−	0.0077	2.17	1.20
Base + FPG + TG + HDL-C + BMI	0.854 (0.818, 0.885)	0.046 (0.014, 0.079)	0.0076	1.42	1.27

ΔAUC was calculated as the AUC of the component-variable model minus that of the matched index model; positive values favor the component-variable model. Differences were calculated from unrounded estimates.

**Figure 6:**
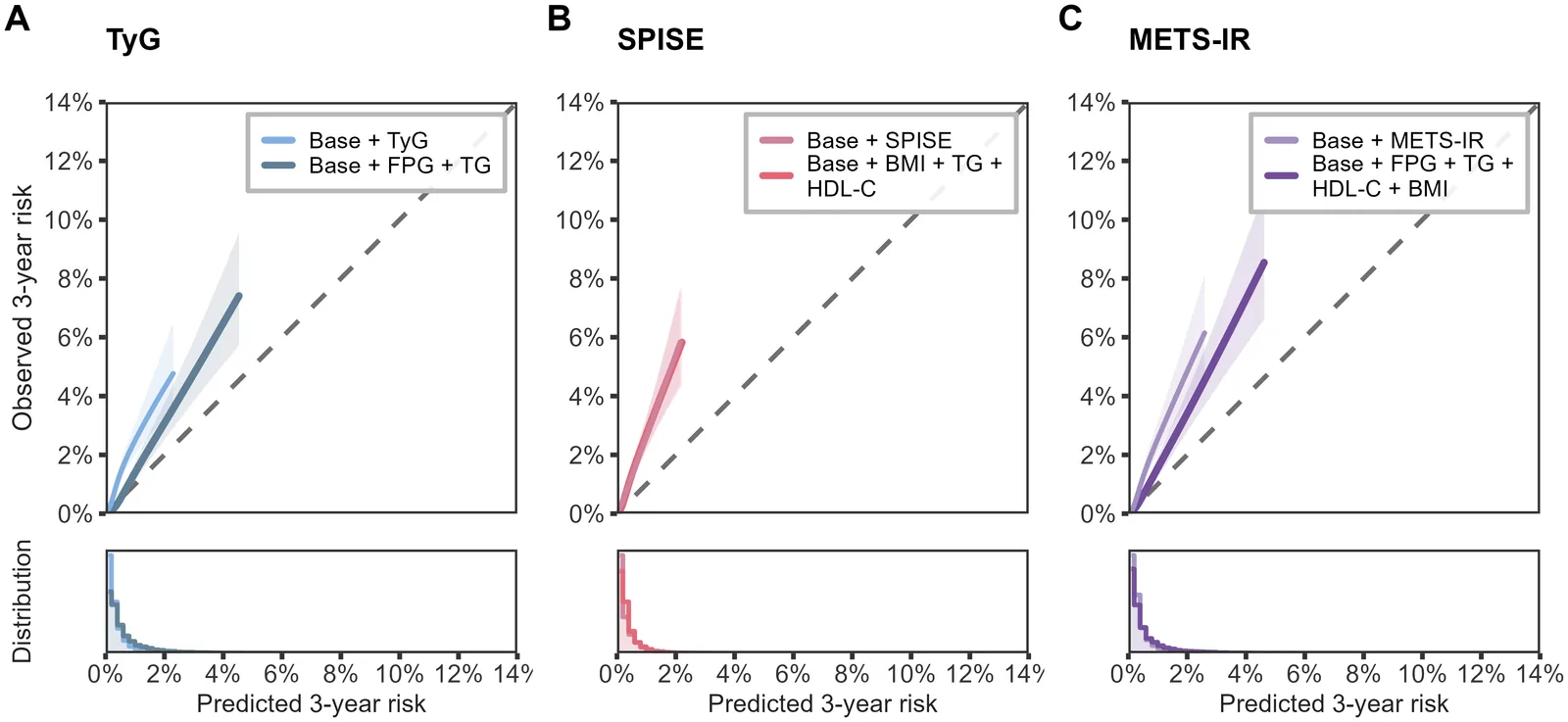
External calibration of the transported index and matched component-variable models at 3 years in NAGALA. **(A)** TyG model and the matched FPG + TG model. **(B)** SPISE model and the matched BMI + TG + HDL-C model. **(C)** METS-IR model and the matched FPG + TG + HDL-C + BMI model. All models included age, sex, SBP and ALT. Solid colored lines show smoothed calibration curves, and shaded areas indicate 95% confidence intervals. The diagonal dashed line represents perfect calibration. The lower panels show the distributions of predicted 3-year risks. All models were developed in RICH and applied to NAGALA without refitting or recalibration.

### 3.5 Contribution of fasting plasma glucose

Adding FPG to the base model increased the optimism-corrected 3-year AUC by 0.081 (95% CI, 0.064–0.098) in RICH. In NAGALA, the corresponding increase was 0.118 (95% CI, 0.077–0.159). Further addition of TyG, SPISE or METS-IR yielded smaller AUC gains in RICH. In NAGALA, only the METS-IR increment was statistically significant after Holm adjustment (**Table 5**).

**Table 5:** Incremental 3-year AUC after adding each index to the base model containing FPG.

Index added	RICH ΔAUC (95% CI)	NAGALA ΔAUC (95% CI)	NAGALA Holm-adjusted *P* value
TyG	0.005 (0.001, 0.009)	0.003 (−0.008, 0.015)	1.000
SPISE	0.013 (0.007, 0.020)	0.005 (−0.017, 0.025)	1.000
METS-IR	0.014 (0.009, 0.020)	0.017 (0.003, 0.031)	0.036

Base included age, sex, systolic blood pressure and ALT. ΔAUC represents the AUC of Base + FPG + index minus that of Base + FPG. RICH estimates were optimism-corrected; NAGALA estimates used frozen RICH models. *P* values were Holm-adjusted across the three NAGALA AUC comparisons; confidence intervals were not multiplicity-adjusted.

When FPG was retained separately in both index and component-variable models, AUC differences narrowed (**Figure 7A**). In RICH, component-variable models had slightly higher AUCs than the SPISE and METS-IR models, while the TyG models showed little difference. In NAGALA, the paired TyG and SPISE models showed no clear AUC differences. The METS-IR model had a higher AUC than its matched component-variable model (component-minus-index ΔAUC, −0.0127; 95% CI, −0.0220 to −0.0038; Holm-adjusted *P* = 0.012).

**Figure 7:**
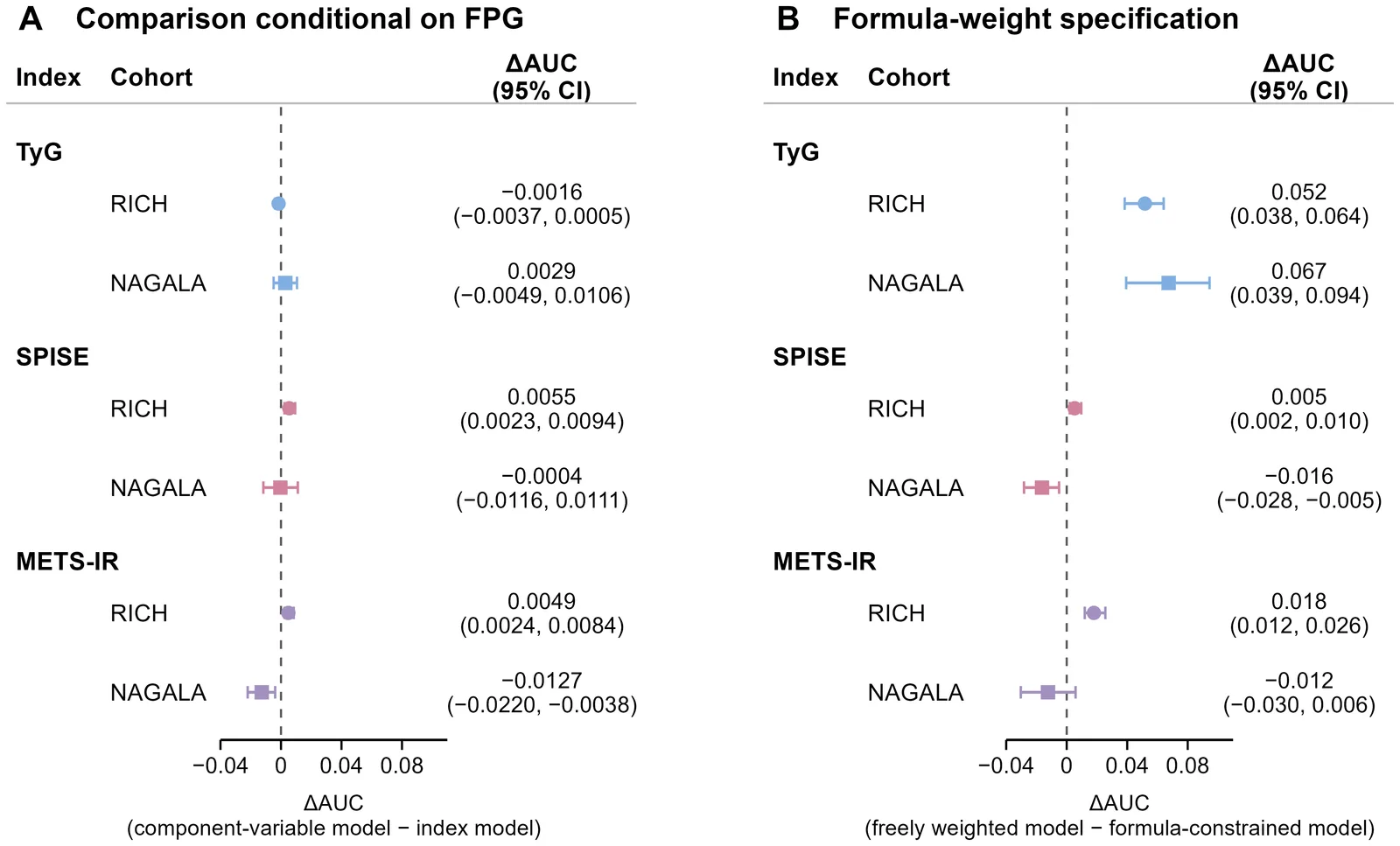
Paired differences in 3-year AUC in RICH and NAGALA. **(A)** Component-variable minus index-model AUC, with FPG retained separately in both models. **(B)** Freely weighted minus formula-constrained model AUC on the TyG, −ln(SPISE) and ln(METS-IR) scales; only outer weights were freed for METS-IR. All models included age, sex, systolic blood pressure and ALT. Points represent paired ΔAUC estimates, and horizontal lines indicate 95% confidence intervals. Circles denote RICH (n = 112,070) and squares NAGALA (n = 15,453). The vertical dashed line indicates no difference in AUC. RICH estimates were optimism-corrected; NAGALA estimates used frozen RICH models. Confidence intervals were not multiplicity-adjusted.

### 3.6 Evaluation of fixed formula weights

In RICH, conventional likelihood-ratio tests showed better fit with freely estimated formula-scale coefficients for all three indices (all Holm-adjusted *P* < 0.001; **Table 6**). Center-clustered Wald tests supported departure from the tested constraints for TyG and METS-IR (both Holm-adjusted *P* < 0.001), but not for SPISE (Holm-adjusted *P* = 0.073; **Supplementary Table S1**). For TyG, the constrained model estimated a common coefficient of 0.944 for ln(FPG) and ln(TG), compared with 8.849 and 0.584 when estimated separately. Freely estimating the coefficients increased the optimism-corrected 3-year AUC by 0.052 for TyG, 0.005 for SPISE and 0.018 for METS-IR.

**Table 6:** Likelihood-ratio tests of formula constraints and fitted component coefficients in RICH.

Index	Formula-scale term	Constrained coefficient	Free coefficient (95% CI)	LRT χ²	df	Holm-adjusted *P* value
TyG	ln(FPG)	0.944	8.849(7.578, 10.120)	821.14	1	<0.001
	ln(TG)	0.944	0.584(0.465, 0.703)			
SPISE	ln(BMI)	3.324	3.922(3.601, 4.242)	56.32	2	<0.001
	ln(TG)	0.497	0.581(0.482, 0.680)			
	ln(HDL-C)	−0.460	0.462 (−0.368, 1.291)			
METS-IR	ln(BMI)	3.528	3.656 (3.300, 4.011)	232.66	2	<0.001
	ln[ln(2FPG + TG)]	3.528	9.857 (9.162, 10.551)			
	ln[ln(HDL-C)]	−3.528	2.443 (−0.434, 5.320)			

Constrained coefficients equal the fitted score coefficient multiplied by the corresponding formula weights. The constrained scores were TyG, −ln(SPISE) and ln(METS-IR); only outer weights were freed for METS-IR. FPG, TG and HDL-C were expressed in mg/dL, and BMI in kg/m². Coefficient confidence intervals used center-clustered standard errors; LRTs were based on conventional partial likelihood. *P* values were Holm-adjusted across the three LRTs.

In NAGALA, the freely weighted TyG model had a higher AUC than its formula-constrained counterpart (free-minus-fixed ΔAUC, 0.067; 95% CI, 0.039–0.094; Holm-adjusted *P* = 0.006). For SPISE, the formula-constrained −ln(SPISE) model had a higher AUC than the freely weighted model (free-minus-fixed ΔAUC, −0.016; 95% CI, −0.028 to −0.005; Holm-adjusted *P* = 0.012). No clear AUC difference was observed between the two models on the ln(METS-IR) scale (**Figure 7B**).

### 3.7 Sensitivity analysis

Among 197,472 RICH participants otherwise eligible for the TyG analysis, 85,402 (43.2%) were excluded from the complete-case cohort because HDL-C was unavailable. Excluded participants were younger (SMD, 0.352), whereas SMDs for the other available baseline characteristics were ≤0.090 (**Supplementary Table S2**). In the expanded samples, component-minus-index ΔAUCs for TyG were 0.051 (95% CI, 0.040–0.062) in RICH and 0.071 (0.038–0.104) in NAGALA, similar to the complete-case estimates.

In the restricted cubic-spline sensitivity analysis, the component-variable models retained higher 3-year AUCs than the corresponding TyG and METS-IR models in NAGALA. The paired AUC differences were 0.074 (95% CI, 0.044–0.107) for TyG and 0.055 (0.022–0.089) for METS-IR (both Holm-adjusted *P* = 0.006). The SPISE model had a slightly higher AUC than its component-variable model (component-minus-index ΔAUC, −0.011; 95% CI, −0.021 to −0.001; Holm-adjusted *P* = 0.046), although this small difference was not observed in the same direction in RICH.

At 2.5 years, component-variable models had higher AUCs than the matched TyG and METS-IR models in both cohorts. The SPISE differences remained small and opposite in direction between cohorts (**Supplementary Figure S2**).

## 4 Discussion

In this study, TyG, SPISE and METS-IR were evaluated for incident diabetes prediction in two East Asian health-screening cohorts. In RICH, all three indices showed clear risk gradients, nonlinear associations with incident diabetes, and similar discrimination when evaluated individually. Comparing each index with a model containing the same component variables provided a different view of their predictive performance. In RICH, component-variable models achieved higher optimism-corrected 3-year AUCs than the corresponding TyG-, SPISE- and METS-IR-based models, with differences of 0.051, 0.009 and 0.059, respectively. The higher discrimination of the component-variable models was retained in NAGALA for TyG- and METS-IR-based comparisons, whereas SPISE and its component-variable model showed similar external performance. These differences narrowed when FPG was retained separately. Freely estimating formula-scale coefficients improved discrimination in both cohorts only for TyG. Taken together, these findings suggest that composite indices remain useful for diabetes risk stratification, while differences from component-variable models may partly reflect the independent contribution of FPG and, particularly for TyG, fixed formula weighting.

These findings align with previous studies showing that non-insulin-based surrogate indices capture clinically relevant metabolic risk. Previous cohort studies have associated higher TyG and METS-IR, and lower SPISE, with subsequent dysglycemia or diabetes (18–20). Previous meta-analysis and cohort studies have also demonstrated nonlinear risk relationships and useful time-dependent discrimination for TyG-related indices and METS-IR (21–23). Our findings therefore reinforce the value of these indices for identifying metabolic risk gradients. The present analysis extends this evidence by addressing a complementary question: how fixed index formulas compare with direct joint modeling of the same routinely measured variables.

The observed differences between the index-based and component-variable models are plausible given how these indices are constructed. TyG represents a logarithmic transformation of the product of FPG and TG, whereas METS-IR combines FPG, TG, HDL-C and BMI using predetermined mathematical relationships. These transformations provide concise summaries of metabolic status, but their coefficients and functional forms were originally developed to estimate insulin sensitivity or insulin resistance rather than specifically optimized for diabetes prediction in a particular population (4–6). By entering the component variables separately, the prediction model can estimate the outcome-specific contribution of each measurement. Adding FPG to the base model produced a large increase in discrimination, indicating its strong predictive contribution. When FPG was retained separately in both the index and component-variable models, the between-model differences narrowed, suggesting that the way FPG was represented may partly explain the original differences. The importance of FPG in these analysis is consistent with a previous RICH study in which FPG alone achieved a higher AUC than TyG or TyG-BMI (10).

The likelihood-ratio tests comparing formula-constrained with freely weighted models provided an assessment of the tested weighting constraints on the specified formula scales, particularly for TyG. Because TyG constrains ln(FPG) and ln(TG) to share the same coefficient, it assumes that these two terms contribute equally to the linear predictor. In contrast, the freely fitted model estimated a much larger coefficient for ln(FPG), consistent with the strong predictive contribution of baseline glycemia in this setting. Thus, the lower discrimination of TyG may partly reflect compression of differently weighted predictors into a fixed equal-weight formula, rather than a lack of prognostic value of TyG itself. These fitted coefficients should not be interpreted as measures of the relative biological importance of glucose and triglycerides, and the same weighting constraint may perform differently for other outcomes or populations. For SPISE and METS-IR, better fit under conventional likelihood-ratio testing did not translate into reproducible external gains, illustrating that better model fit in the development cohort does not necessarily imply better prediction in an external population (24). For SPISE, inference concerning the weighting constraints was sensitive to variance estimation and center deletion (**Supplementary Material**). This finding warrants caution, particularly because the center-clustered Wald tests relied on an asymptotic approximation with only 15 centers.

This interpretation should not be taken to diminish the established value of composite insulin-resistance and sensitivity indices. Their intended role differs from that of an outcome-specific multivariable prediction model (25). A composite index provides a reproducible single-number summary that can facilitate epidemiological comparisons and metabolic risk stratification across populations (26, 27). In contrast, a component-variable model estimates the risk of a defined outcome over a specified time horizon. Predictive performance should therefore be considered in the context of the intended use of these indices. However, when a concise surrogate measure of insulin resistance or sensitivity is required, these indices remain practical and informative. The comparison involving SPISE illustrates this distinction particularly well. The component-variable model showed a small AUC improvement over SPISE in RICH, but no clear difference was observed in NAGALA. Before FPG was added separately, neither the SPISE model nor its matched component-variable model included FPG, unlike the corresponding TyG and METS-IR models. Their similar external performance may suggest that SPISE retains much of the predictive information contained in BMI, TG, and HDL-C in a more parsimonious model form. Nevertheless, because the external confidence interval spanned modest differences in both directions, these findings should not be interpreted as demonstrating formal equivalence.

The validation results also distinguish risk ranking from estimation of absolute risk. The higher discrimination of the TyG and METS-IR component-variable models was broadly reproduced in NAGALA. Similar findings at 2.5 years, with matched splines, and after allowing time-varying coefficients for ALT and, where included separately, HDL-C suggest that these comparisons were robust to prediction horizon, functional form, and the assessed departures from proportional hazards. Meaningful external evaluation, however, requires assessment of calibration as well as discrimination (28–30). All transported models underestimated 3-year diabetes risk in NAGALA, with O/E ratios up to 2.52 and calibration slopes above 1 indicating somewhat insufficient dispersion. Baseline FPG and other predictor distributions differed between cohorts, and NAGALA additionally used HbA1c for diabetes ascertainment; both may have contributed to the observed miscalibration. Differences in follow-up and censoring may also have affected the precision of fixed-horizon IPCW estimates. These influences cannot be separated in the present data. Accordingly, the NAGALA results support external reproducibility of the relative TyG and METS-IR comparisons, but not direct use of the RICH-derived probabilities without recalibration. Brier scores differed little among models, which should be interpreted in the context of the low 3-year event rate rather than as evidence of equivalent prediction. Net benefit depends on both calibration and clinically meaningful risk thresholds (31). Because no specific intervention-linked decision threshold was prespecified and the transported probabilities were not recalibrated, the decision curves are presented in the **Supplementary Material** and should not be interpreted as establishing clinical utility.

The study has several strengths. It used two large, independent cohorts from different East Asian populations and preserved the separation between model development and external validation. All standardization parameters, regression coefficients and baseline risk estimates were transported to NAGALA without refitting or recalibration. The matched comparisons used the same participant samples and the same base model within each index family, reducing the possibility that differences were caused by unequal populations or covariate adjustment. The analysis also considered association, nonlinearity, discrimination, overall prediction error, calibration and net benefit, rather than relying on a single performance measure. Additional analyses of FPG contribution and formula constraints helped assess whether the observed index–component differences were related to independent FPG modeling or fixed weighting. Bootstrap correction, leave-one-center-out validation and participant-level paired bootstrap comparisons further strengthened the evaluation.

Several limitations should be considered. First, both cohorts comprised East Asian adults undergoing routine health examinations, which may limit generalizability to populations with different ethnic backgrounds, healthcare settings and baseline risk profiles. Second, substantial HDL-C missingness in RICH may have introduced selection bias, although the expanded TyG analysis yielded similar results. Third, only 101 diabetes events occurred within 3 years in NAGALA, limiting the precision of external performance estimates, while differences in follow-up and censoring may also have affected the precision of fixed-horizon estimates. Fourth, diabetes ascertainment was not fully harmonized: both cohorts used follow-up FPG, whereas NAGALA additionally included HbA1c, and the public NAGALA data did not permit identification of events ascertained by FPG alone. Finally, the datasets did not distinguish diabetes subtypes. Future studies should evaluate these findings in ethnically and clinically diverse populations, using cohorts with more complete biomarker data and sufficient numbers of events at the prespecified prediction horizon.

## 5 Conclusion

In conclusion, TyG, SPISE, and METS-IR were associated with incident diabetes, and demonstrated moderate discriminatory ability in two independent East Asian health-screening cohorts. Component-variable models showed higher discrimination in the TyG and METS-IR comparisons, whereas the small SPISE difference was not reproduced externally. These differences were attenuated when FPG was retained separately in both models. Freely estimating the formula-scale coefficients improved discrimination over TyG in both cohorts, whereas the corresponding gains for SPISE and METS-IR were not reproduced externally. Our findings suggest that composite indices may remain useful for metabolic risk characterization and epidemiological stratification, but their clinical application for individualized diabetes prediction should not be assumed to outperform models using routinely available component variables. Future prediction models should prioritize direct evaluation of incremental value, calibration, and external validity before recommending simplified composite indices for clinical prediction.

## Data Availability

Publicly available datasets were analyzed in this study. This data can be found here: https://doi.org/10.5061/dryad.ft8750v; https://doi.org/10.5061/dryad.8q0p192.
