# Supplementary material for: Composite indices of insulin resistance and sensitivity versus matched component-variable models for incident diabetes prediction
Source: Front Endocrinol (Lausanne). 2026 Oct 8;17:1957473. doi: 10.3389/fendo.2026.1957473 (PMC13686858; doi:10.3389/fendo.2026.1957473)
Supplement: Supplementary file 1 [file SupplementaryFile1.pdf]

Supplementary Materials

Supplementary tables

Table S1. Sensitivity of formula-constraint tests to inferential method in RICH.

Index	Conventional LRT	Ordinary Wald	Center-clustered Wald
TyG	<0.001	<0.001	<0.001
SPISE	<0.001	<0.001	0.073
METS-IR	<0.001	<0.001	<0.001

Values are Holm-adjusted *P* values across the three indices, separately for each test method. All tests in this table used the same 112,070 RICH participants and 1,425 events during full follow-up. LRT, likelihood-ratio test. SPISE inference varied across center-deletion analyses. Omitting the largest center, which contributed 36.0% of full-follow-up events, yielded Holm-adjusted *P* values of 0.222, 0.223 and 0.508 for the LRT, ordinary Wald and center-clustered Wald tests, respectively. For TyG and METS-IR, all three tests remained significant after every center omission (all Holm-adjusted $P < 0.001$).

Table S2. Characteristics of RICH participants included in and excluded from the complete-case cohort.

Characteristic	Included (n = 112,070)	Excluded (n = 85,402)	SMD
Age, years	43.67 ± 12.76	39.36 ± 11.70	0.352
Sex, n (%)			0.048
Female	52,392 (46.7%)	37,904 (44.4%)	–
Male	59,678 (53.3%)	47,498 (55.6%)	–
BMI, kg/m²	23.27 ± 3.27	23.02 ± 3.35	0.073
SBP, mmHg	118.97 ± 16.44	118.22 ± 15.79	0.046
ALT, U/L	18.00 (13.00, 27.00)	17.80 (12.60, 27.00)	0.014
FPG, mmol/L	4.89 ± 0.54	4.84 ± 0.55	0.090
TG, mmol/L	1.10 (0.75, 1.63)	1.00 (0.70, 1.52)	0.080
HDL-C, mmol/L	1.38 ± 0.30	–	–
Follow-up, years	2.99 (2.13, 3.94)	3.01 (2.23, 3.97)	0.066
Diabetes events during follow-up, n	1,425	758	–
Diabetes events by 3 years, n	478	287	–
3-year cumulative incidence, % (95% CI)	0.63 (0.57, 0.69)	0.48 (0.42, 0.54)	–

Supplementary figure

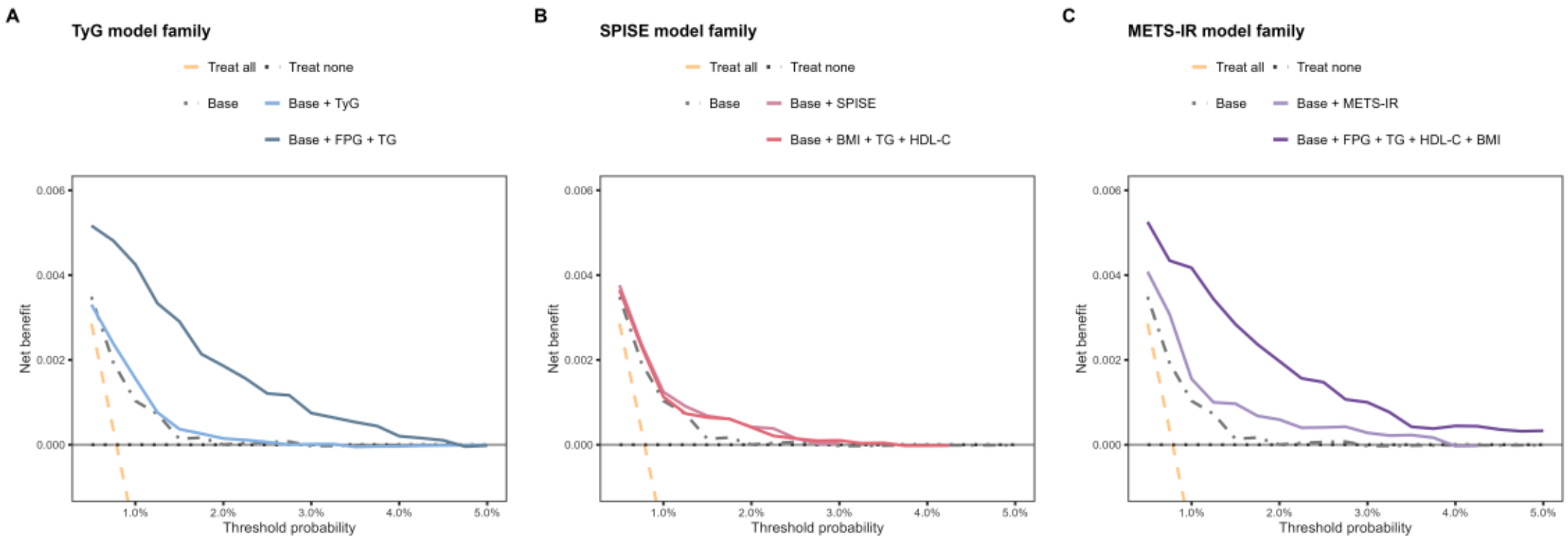

Figure S1. Decision-curve analysis of the transported index and component-variable models in NAGALA. (A) TyG and FPG + TG. (B) SPISE and BMI + TG + HDL-C. (C) METS-IR and FPG + TG + HDL-C + BMI. Each panel includes the base, index and matched component-variable models, alongside the treat-all and treat-none strategies. All models included age, sex, systolic blood pressure and ALT. Net benefit was evaluated at 3-year risk thresholds of 0.5%–5.0%. The analyses were exploratory because the transported risks were not recalibrated and no specific intervention threshold was prespecified.

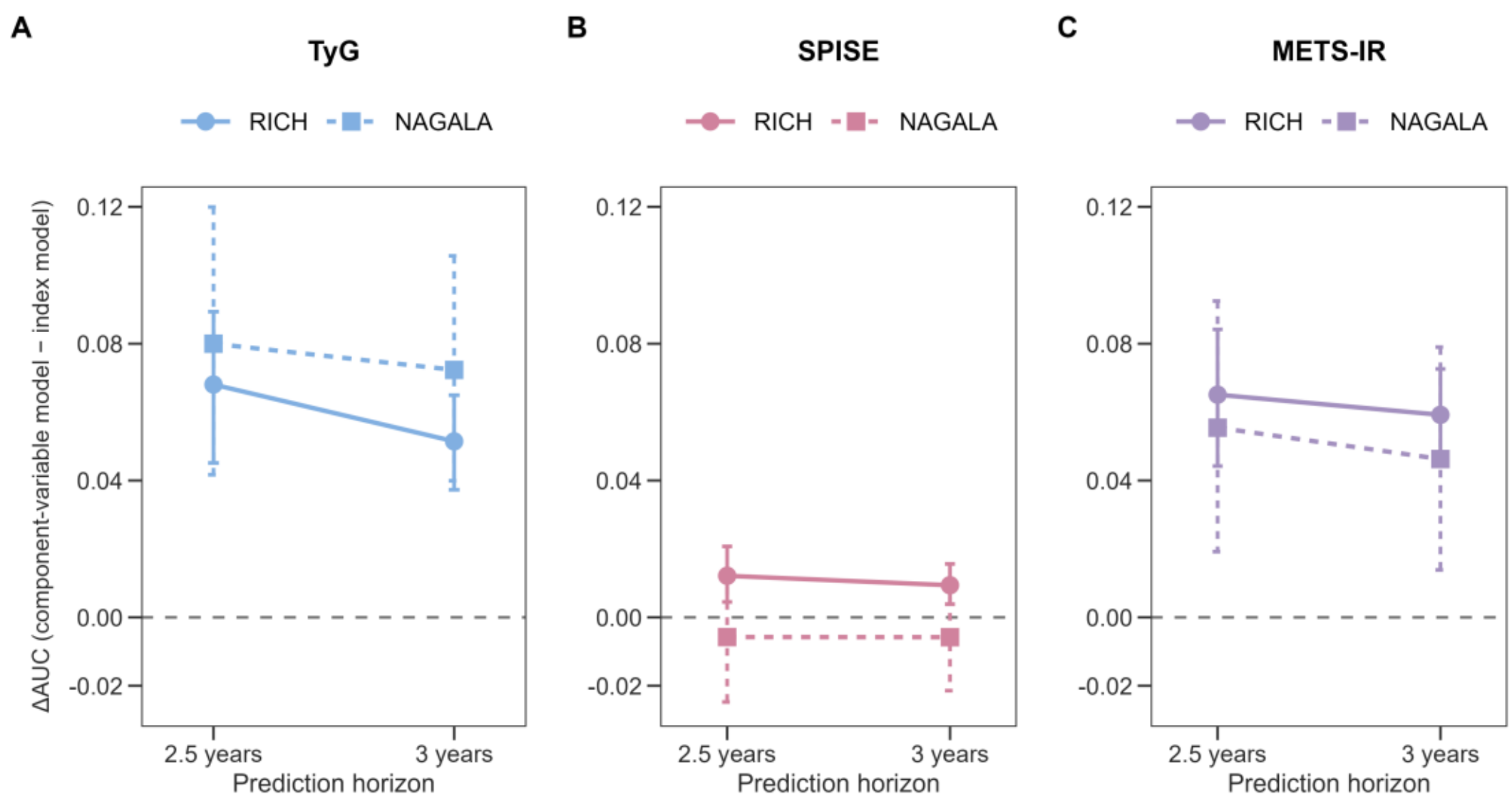

Figure S2. Paired differences in AUC between matched component-variable and index models at 2.5 and 3 years in RICH and NAGALA. (A) TyG; (B) SPISE; (C) METS-IR. Points

represent component-variable minus index-model AUC, and vertical error bars indicate 95% confidence intervals. All models included age, sex, systolic blood pressure and ALT. Circles denote RICH (n = 112,070) and squares NAGALA (n = 15,453). RICH estimates were optimism-corrected; NAGALA estimates used frozen RICH models. Confidence intervals were not multiplicity-adjusted.
